# Gut Microbiome: A Brief Review on Its Role in Schizophrenia and First Episode of Psychosis

**DOI:** 10.3390/microorganisms10061121

**Published:** 2022-05-29

**Authors:** Konstantinos Tsamakis, Sofia Galinaki, Evangelos Alevyzakis, Ioannis Hortis, Dimitrios Tsiptsios, Evangelia Kollintza, Stylianos Kympouropoulos, Konstantinos Triantafyllou, Nikolaos Smyrnis, Emmanouil Rizos

**Affiliations:** 1 Second Department of Psychiatry, Attikon University General Hospital, National and Kapodistrian University of Athens, 12462 Athens, Greece; sgalinaki@gmail.com (S.G.); ale.vag77@yahoo.gr (E.A.); hortis.giannis@gmail.com (I.H.); kollintzaevangelia@gmail.com (E.K.); s.kympouropoulos@gmail.com (S.K.); smyrnis@med.uoa.gr (N.S.); erizos@med.uoa.gr (E.R.); 2 Institute of Psychiatry, Psychology and Neuroscience (IoPPN), King’s College London, London SE5 8AB, UK; 3 Institute of Medical and Biomedical Education, St George’s, University of London, London SW17 0RE, UK; 4 Neurology Department, Democritus University of Thrace, 68100 Alexandroupolis, Greece; tsiptsios.dimitrios@yahoo.gr; 5 Hepatogastroenterology Unit, 2nd Department of Propaedeutic Internal Medicine, Medical School, Attikon University General Hospital, National and Kapodistrian University of Athens, 12462 Athens, Greece; ktriant@med.uoa.gr

**Keywords:** microbiota, gut, brain, axis, psychopathology, psychosis, first episode, schizophrenia, microbiome, alterations

## Abstract

There is a growing body of evidence highlighting the role of gut microbiota as a biological basis of psychiatric disorders. The existing literature suggest that cognitive and emotional activities can be influenced by microbes through the microbiota–gut–brain axis and implies an association between alterations in the gut microbiome and several psychiatric conditions, such as autism, depression, bipolar disorder and psychosis. The aim of this review is to summarise recent findings and provide concise updates on the latest progress of the role of gut microbiota in the development and maintenance of psychiatric symptoms in schizophrenia and the first episode of psychosis. Despite the lack of consistent findings in regard to specific microbiome changes related to psychosis, the emerging literature reports significant differences in the gut microbiome of schizophrenic subjects compared to healthy controls and increasingly outlines the significance of an altered microbiome composition in the pathogenesis, development, symptom severity and prognosis of psychosis. Further human studies are, however, required, which should focus on identifying the drivers of microbiota changes in psychosis and establish the direction of causality between psychosis and microbiome alterations.

## 1. Introduction

The human body consists of a number of microbial environments that are predominantly made up of bacteria, but also includes archaea, fungi, protists and viruses [1]. The intestinal or gut microbiome is the largest ecological community of the approximately 3.9 × 10^13^ bacteria in the human body, nearly equal with the number of adult human cells [2], most of which are located in the distal gut and its genetic material outnumbers human DNA by 10-fold [3]. Despite the terms ‘microbiome’ and ‘microbiota’ being sometimes used interchangeably [4], they have subtle differences: microbiome describes the entire environment, including the microorganisms, their genomes (i.e., genes), and the surrounding environmental conditions, whereas microbiota is usually used to describe the assemblance of all the microorganisms within a specific, defined environment [5].

It has been shown that the gut microbiome plays a vital role in modulating a bidirectional communication between the central nervous system and gut bacteria [6]. Reciprocal signalling between the gut and the brain is regulated at the neural, immune and endocrine level [7]. These pathways interrelate with the gut microbiome and, together, they comprise the microbiota–gut–brain axis, which is defined by the bidirectional communication between the digestive system and the nervous system, involving the central nervous system, the autonomic nervous system, and the enteric nervous system [8]. This ‘microbiota–gut–brain’ axis regulates several important functions, including immunity, digestion, metabolism, satiety and stress reactions [9,10]. Gut bacteria affect these processes through their ability to synthesise neurotransmitters, the modulated activation of the immune system and by producing metabolites that may have neuroactive properties [11]. It has been shown that the gut microbiome modulates the central nervous system primarily through neuroimmune and neuroendocrine mechanisms involving the vagus nerve; the communication is mediated by numerous microbially derived molecules that include short-chain fatty acids, secondary bile acids and tryptophan metabolites, some of which may cross the intestinal barrier, enter systemic circulation and possibly cross the blood–brain barrier [12]. Consequently, alterations in gut microbiome composition may, in turn, influence psychopathology [13].

Animal models have demonstrated strong, though indirect, evidence for a contributory role of intestinal microbiome in psychiatric symptomatology [14]. In animal models, stress, which underlies most psychiatric conditions, has been shown to influence the function and composition of the gut microbiome and host metabolism. In addition, altering the gut microbiome composition with faecal microbiota transplantation (FMT) can directly moderate metabolic function in both animals and humans and result in behavioural changes in rodents [15]. In humans, it has been suggested that microbiome-induced alterations in neural signs and in the tryptophan metabolism in the gut, resulting in a microbiome-induced reduction in plasma tryptophan concentration (which affects the brain synthesis of serotonin and melatonin), could secondarily affect brain physiology and function [15,16].

The possible role of the gut microbiome in the pathophysiology of psychiatric conditions has received increasing attention in recent years [17]. In this context, probiotic supplementation has been shown to have sustained effects on gut microbiota [18]. Probiotics are live microorganisms, which upon ingestion in adequate amounts can exert health benefits to the host [19]. Probiotic supplements contain living beneficial bacteria, typically belonging to the genera *Lactobacilli* and *Bifidobacteria* [20]. Although in a strain-dependent manner, primarily due to the differential capability of probiotic strains in colonising the human gut [21], the anti-inflammatory and immune-modulatory properties shown by probiotics [22,23] have gained immense notice in the recent years, with such microorganisms being used for numerous clinical applications. Although certain studies have demonstrated their beneficial psychological effects, such as anxiolytic-like activity [24], and the literature has outlined the great potential positive impact of probiotics and emerging psychobiotics on mental disorders [23], the outcomes of treatment trials with probiotics have been somewhat insignificant, both for depressive symptoms [25] and psychotic symptoms [26]. On a similar note, although ‘gut biotics’ [27], such as prebiotics and synbiotics, could be potentially helpful in treating mental health disorders and particularly depression and anxiety [27,28,29], recent meta-analytic data indicate that there is not yet strong enough evidence to support the inclusion of probiotic, prebiotic and symbiotic supplements in treatment guidelines for depression [30]. At the same time, research around paraprobiotics, proteobiotics and postbiotics is still very limited [27], with a need for much more data from randomised controlled trials.

To date, there is ample available evidence proposing an association between alterations in gut microbiome and several psychiatric disorders [16]. For example, microbial dysbiosis (disruption of the gut ecosystem caused by an imbalance in the microbiome) has been observed in autism spectrum disorders, depression, bipolar disorder, post-traumatic stress disorder (PTSD), attention deficit hyperactivity disorder (ADHD), anorexia nervosa and schizophrenia [13,15]. Most studies on the relationship between microbiome and psychiatric symptoms have been conducted in autism spectrum disorders (ASD), where gastrointestinal symptoms, increased intestinal permeability and microbiome alterations (such as elevated levels of *Clostridium* bacteria) have been frequently reported in individuals with ASD [15,16], along with increased levels of lipopolysaccharides and pro-inflammatory cytokines (such as interleukin-6 (IL-6)) [13]. In depression and anxiety, a recent systematic review showed inconsistent findings in regards with microbiome alpha and beta diversity, but the differences in bacterial taxa indicated a higher abundance of proinflammatory taxa (e.g., *Enterobacteriaceae* and *Desulfovibrio*), and lower short-chain fatty acid producing-bacteria (e.g., *Faecalibacterium*) in these mood disorders [31]. In bipolar disorder, a small number of studies have shown alterations in the levels of gut bacteria with anti-inflammatory properties, such as a higher abundance of *Flavonifractor* and a lower amount of *Faecalibacterium* [16]; given the role of inflammation in this mental disorder [32], this could suggest an involvement of the microbiome in the pathophysiology of bipolar illness [13].

Dysbiosis and alterations in the composition and function of the gut microbiome are frequently found in the gut of patients with schizophrenia [33,34], while an increased risk of schizophrenia has been associated with numerous factors that relate to the gut microbiome [13]. Schizophrenia, a leading cause of disability worldwide with a lifetime prevalence of about 1%, is a highly heterogenous disorder where the interactions between environmental and genetic factors play a significant role [35]. Early intervention during the first episode of psychosis (FEP) is particularly important, so as to minimise the duration of untreated psychosis, improve treatment response and prevent relapse [36]. The aim of this review is to summarise the latest updates on the role of gut microbiome in the development, maintenance and severity of psychiatric symptoms in the first episode of psychosis and schizophrenia.

## 2. Gut Microbiome and Schizophrenia

### 2.1. Pathophysiological Mechanisms

High rates of comorbidity with autoimmune and gastrointestinal conditions, systemic low-level inflammation and increased intestinal permeability have all been reported in schizophrenia, suggesting the unquestionable involvement of the gut microbiome [37]. Additionally, strong evidence suggests that the immune system plays a role of utmost importance in the pathogenesis and development of schizophrenia [38]. Considering that (a) the gut is the most dynamic immunological environment in the body, (b) gut microbiome colonisation early in life is critical for the optimal development of the immune system [39], and (c) that dysbiosis of the intestinal ecosystem may affect immune responses [40], dysfunctions such as chronic inflammation and oxidative stress, which have been implicated in schizophrenia, are proposed to be, at least in part, associated with changes in the microbiome [14]. These include the microbiome’s mediation in the regulation of pro-inflammatory cytokines, which may, in turn, influence the pathophysiology of mental disorders [16]; for example, increased IL-6, IL-1β and IL-2R concentrations are reported in chronic schizophrenic patients [41].

In addition, two recent studies indicated a causative role of dysbiosis in the pathogenesis of schizophrenia [34] and provided a mechanistic explanation through the modulation of tryptophan–kynurenine metabolism [42] (which has been demonstrated to be abnormal in schizophrenia [43]) and glutamatergic neurotransmission metabolism [44]. More specifically, Zhu et al. recolonised specific pathogen-free mice with faecal gut microbiota from schizophrenic patients and healthy controls and found the mice’s gut differentially enriched in the two groups of mice [42]. They also found significant changes in the tryptophan metabolism pathway and neurotransmitter levels, with microbiome derived from schizophrenic faeces disturbing the tryptophan metabolism pathway in both the peripheral and central nervous systems in mice. More specifically, in ‘schizophrenic’ mice, the Kyn–Kyna pathway of tryptophan catabolism was markedly increased, whereas the serotonin pathway of tryptophan catabolism was reduced [42]. An independent study from the same research group showed that microbiome alterations may potentially be involved in the onset and pathophysiology of schizophrenia through the modification of metabolic pathways in the microbiota–gut–brain axis [44]. More specifically, the recipient mice of the gut microbiome of schizophrenic patients displayed altered amino acid and lipid metabolism, along with disruptions in the glutamate–glutamine–GABA cycle and decreased brain glutamate [44], which has been implicated in the pathophysiology of schizophrenia [45]. Decreased glutamate could be potentially associated with NMDA (N-methyl-D-aspartate) receptor hypofunction in schizophrenia [46], while reductions in dendrite arborisation, spine density and the synaptophysin expression of glutamate neurons have also been noted across the frontal and temporal regions [46]. In addition, a recent systematic review highlighted numerous functional metabolic pathways that were different in schizophrenia compared to the controls, such as vitamin B6, fatty acid, starch and sucrose, cysteine, methionine and linoleic acid metabolism, as well as the degradation of some xenobiotics (e.g., foreign substances to the body or ecological system) [47]. These differential metabolic pathways were associated with specific taxa, *Blautia*, *Coprococcus*, and *Roseburia*, which were negatively correlated with the vitamin B6, taurine and hypotaurine metabolic pathways, and positively correlated with the methane metabolic pathway [47,48]. Finally, recent findings suggest that there are significant correlations between the properties of the gut microbiome and alterations in brain structure and function in schizophrenic patients [49,50]. More specifically, the indexes of alpha diversity in schizophrenia were positively associated with the regional homogeneity indexes of the bilateral calcarine cortex, bilateral lingual gyrus, left superior occipital cortex and right superior parietal cortex [49], while specific schizophrenia-associated microbiota was correlated with the right middle frontal gyrus volume, which appears to be abnormal in schizophrenic patients [50].

### 2.2. Microbiome Diversity

The number of empirical studies on the gut microbiome in people with schizophrenia is limited [14]. To date, studies exploring microbiome differences between healthy controls and subjects with schizophrenia have reported significant differences in the abundance of specific taxa and species composition between the two groups [20,49]. In a recent study by Li et al., the relative abundance of *Ruminococcus* and *Roseburia* was significantly lower at the genus level, whereas the abundance of *Veillonella* was significantly higher in schizophrenic patients compared to controls [49]. Nearly all studies have investigated potential alterations and differences in the alpha and beta diversity of the microbiome. Alpha diversity, which is overall thought to represent a marker of “good” health status [44], depicts within-group diversity (showing ‘how many different species were found’”, i.e., how many different bacteria exist in a healthy individual), while beta diversity represents diversity between groups (i.e., how different was the diversity of bacteria between healthy controls compared to diseased individuals) [51]. A very recent paper reported that most studies to date have found no changes in alpha diversity between the schizophrenia and control groups, while showing significant differences in beta-diversity [52], in concordance with a recent systematic review that outlined that beta-diversity was consistently reported to be different between schizophrenia and controls [47]. These findings (i.e., no changes in alpha diversity and significant differences in beta diversity) are also in line with a previous study [53], which showed that *Proteobacteria* were reduced in patients with schizophrenia, and also with another earlier study, which indicated different microbiome beta diversity in chronic schizophrenic patients compared to controls, with *Proteobacteria* and *Fusobacteria* significantly more abundant and *Firmicutes* reduced in schizophrenic patients [48].

In contrast, a study by Ma et al. [50] and a study by Zheng et al., which investigated the microbiome in both humans (schizophrenic and controls) and germ-free mice receiving schizophrenia microbiome faecal transplants, showed reduced alpha diversity [44]. This study also revealed alterations in beta diversity, with specific microbes (*Aerococcaceae*, *Bifidobacteriaceae*, *Brucellaceae*, *Pasteurellaceae* and *Rikenellaceae*) making it possible to discriminate schizophrenic patients from healthy controls. When the investigators transplanted faecal microbiome from patients with schizophrenia to germ-free mice, their gut microbiome was significantly different to that of the control mice, with the most altered bacterial families being *Aerococcaceae* and *Rikenellaceae* [44], similar to changes found in subjects with schizophrenia [51]. In general, animal studies that have used translationally valid models for schizophrenia have largely led to inconsistent conclusions regarding microbiome alterations in schizophrenia; however, there is some convergence in reporting reduced levels of the phylum *Proteobacteria* and increased *Actinobacteria* and *Bacteroidetes* [51].

### 2.3. Mediating Factors

The Human Microbiome project revealed the microbial taxa complexity in the human gut and also highlighted the highly individualised microbiome composition due to inheritance, diet and environmental factors [54]. Given the complex relationship between the gut microbiome and schizophrenia, a number of mediating variables should be taken into consideration. Confounding factors, such as gender and geographical location, do not appear to play a significant role, especially since location is usually controlled for in the included studies [51]. In contrast, age might significantly contribute to alterations in the gut microbiome. For instance, gastrointestinal changes seen in aging are characterised by a reduction in diversity, a decline in beneficial bacteria, enrichment of pro-inflammatory microbes and immune system changes related to chronic inflammation [55,56]. A recent literature review that reported frequent contradictory findings regarding microbiome alterations in schizophrenic patients concluded that factors such as age may influence microbiome changes in schizophrenia [16]. Early life events, such as preterm birth, have also been proposed to play a role in the relationship between gut microbiome and schizophrenia. Preterm birth increases the risk for developing psychosis potentially through impaired neurodevelopment. More specifically, the infant’s immature nervous system is particularly vulnerable to neonatal brain injury, which can alter the programmed corticogenesis of the developing brain, while long-lasting structural brain changes have been detected after very preterm birth, including alterations in the temporal and frontal cortices, hippocampus and thalamus [57]. Given that preterm birth has been linked to microbiome alterations (i.e., increased abundance of *Proteobacteria* and a lack of *Bifidobacterium* and *Lactobacillus*) [58], it has been proposed that early life events may affect the development of psychosis later in life, perhaps through microbiome-induced brain changes [51]; however, much more research is required in order to confirm this and establish a causal link.

Factors related to lifestyle changes in psychosis, such as unhealthy diet [59] and smoking [60], have been proven to impact the composition of gut microbiome in schizophrenia [52]; however, it is unclear whether these alterations are significant, partly due to the lack of reporting of smoking status and dietary habits in the majority of the studies [51]. The same stands for exercise, which is known to be significantly reduced in schizophrenia [61]. Although one study has reported that physical activity influences the gut microbiome composition in the context of schizophrenia [62], there is currently no consistent evidence on this [51]. Finally, another factor is treatment with antipsychotics, with recent literature suggesting that antipsychotics could influence the microbiome composition and could potentially confound changes in microbiome of people with schizophrenia [51,52].

In conclusion, the existing literature indicates that there are significant microbiome changes in schizophrenia, as indicated by the reported alterations in beta diversity. A recent systematic revies estimated that around 130 taxa were significantly different between schizophrenia and controls [47]. Overall, studies have demonstrated differential changes for all major phyla in patients with schizophrenia compared to healthy controls, including *Proteobacteria*, *Firmicutes*, *Bacteroidetes*, *Actinobacteria* and *Fusobacteria* [51]. These microbiome alterations appear to be influenced by a number of demographic, environmental and lifestyle factors. Additional research is required in order to identify consistent microbiome changes in psychosis and factors associated with them and establish potential causal links.

### 2.4. Association with Clinical Characteristics of Schizophrenia

The recent literature indicates that changes in microbiome, although highly variable, are not sporadic and that specific microbiome variations can be related to certain clinical features of schizophrenia [52]. In animal studies, hyperactivity has been linked to an elevation in *Lachnospiraceae* and *Clostridiaceae* and at genus level to an increase in *Roseburia*, *Clostridium* and *Odoribacter* [63]. In another study, socially isolated animals showed increased locomotor activity in relation to reduced *Clostridales*, which has also been negatively correlated with cognitive performance [64]. In addition, taxa belonging to the order *Clostridales*, at the family level *Ruminococcaceae*, were positively correlated to anxiety-like behaviours, in contrast to the negative correlation between *Bacillales* and anxiety-like behaviours [64]. Depressive symptoms were linked to an elevated abundance of *Bacteroides* [51], while *Verrucomicrobia* was positively correlated with self-reported mental well-being [47].

Alterations in gut microbiome have also been associated with severity and type of schizophrenia symptoms, such as negative symptoms, and overall global functioning. The most consistent findings of associations between gut microbiome alterations and schizophrenia characteristics are in relation to negative symptom severity and overall symptom severity [52]. A recent systematic review reported that, although findings for *Ruminococcaceae* were mixed, the abundance of *Ruminococcaceae* was reported to be linked to a reduced severity of psychotic symptoms (and specifically negative symptoms) and improved self-reported physical health, implying that this taxon may be protective [47]. In Zheng’s study, *Veillonellaceae* OTU191 were negatively associated with scores of the Positive and Negative Syndrome Scale (PANNS), whereas *Bacteroidaceae* OTU172, *Streptococcaceae* OTU834 and two *Lachnospiraceae* OTUs (477 and 629) were positively associated with PANSS scores [44]. Gut microbiome changes have also been linked to positive symptoms, overall function, likelihood of remission, cognition, treatment resistance, violence, later onset of disease and well-being, but these findings have not been replicated [52].

The mechanisms through which certain bacteria influence the development of specific traits in schizophrenia are not fully understood. Studies involving probiotic supplementation in schizophrenia have shed some light on this; however, their results are inconsistent [20]. In a randomised controlled trial, when a probiotic supplement containing *Lactobacilli* and *Bifidobacterium bifidum* was given (along with vitamin D) to schizophrenic subjects, it decreased CRP levels and enhanced the total antioxidant capacity of the plasma, which led to a significant improvement in the general and total PANSS scores, indicating reduced inflammation [65]; however, it was unclear which component caused the improvement. In another open-label probiotic study of schizophrenia, recipients of *Bifidobacterium breve A-1* had increased levels of IFN-γ, IL-22, IL-1R, IL-10 and reduced levels of TNF-α, which were associated with improved PANSS and anxiety/depression scores [66]; however, the results were difficult to interpret as some of these molecules have both anti- and pro-inflammatory properties [20].

## 3. Gut Microbiome and First Episode of Psychosis

### 3.1. Alterations in Microbiome Composition

Previous research on gut microbiome and first episode of psychosis (FEP) has shown taxonomic differences with approximately 25 taxa significantly different among FEP patients, high-risk patients and non-psychiatric comparison groups [47]. The family *Lactobacillaceae* presented the strongest alterations and constituted the taxa most strongly increased in FEP [62]. A study by He et al. (2018) focused on gut microbiome in relation to the prodromal stage of schizophrenia and included ultra-high-risk subjects for schizophrenia, high-risk subjects and healthy controls. The main finding of this research was that levels of *Clostridiales*, *Prevotella* and *Lactobacillus ruminis* in gut microbiome and choline concentrations in the anterior cingulate cortex were elevated in ultra-high-risk subjects compared to the other two groups [67]. Another study in first episode drug-naïve patients found significant abnormalities in the microbiome composition (lower numbers of faecal *Bifidobacterium* spp., *Escherichia coli*, *Lactobacillus* spp. and higher numbers of faecal *Clostridium coccoides*) of psychotic patients compared to healthy controls [68]. A small cross-sectional Chinese study (2020) in FEP patients found altered composition in the gut microbiome of the drug-naïve, psychotic subjects, characterised by a relative reduction in alpha diversity and increased abundance of the harmful phylum *Proteobacteria* [69]. Ma et al. (2020) found marked changes in the gut microbial composition of first-episode patients in certain taxa, such as *Christensenellaceae*, *Enterobacteriaceae*, *Pasteurellaceae*, *Turicibacteraceae* at the family level and *Escherichia* at the genus level, compared to the controls [50]. Finally, another study by Zhu et al. (2020), which examined medication-free psychotic patients, found that they had a higher alpha diversity and higher beta diversity in their gut microbiome compared to controls [70]. Table 1 summarises the changes in microbiome composition and possible pathophysiological mechanisms involved in the first episode of psychosis and schizophrenia.

### 3.2. Pathophysiological Mechanisms in FEP and Association with Clinical Features and Prognosis of Psychosis

Although microbiome-related pathophysiological mechanisms in FEP are not substantially different than those in chronic schizophrenia, it is important to highlight the relevant existing evidence. This is because the functional impairment in psychosis occurs most rapidly during this early ‘critical’ period, which can alter the patient’s future prognosis, treatment level and morbidity [71], while the early treatment of these drug-naïve, first-episode psychotic patients offers the greatest promise for symptomatic and functional recovery. Changes in the gut microbiome during the first episode of psychosis may be the basis for microglial activation and subsequent disruption of the membrane metabolism in the brain [67]. Additionally, lower serum tryptophan levels and higher kynurenic acid (KYNA) levels have been observed in medication-free psychotic patients implying that, in psychosis, altered gut microbiota may be associated with changes in serum levels of tryptophan and KYNA [70]. Interestingly, following risperidone treatment in FEP subjects, significant changes in certain faecal bacteria were noted, which were possibly associated with antipsychotic-medication-induced metabolic changes [68]. Distinct changes in microbiome following antipsychotic treatment were also reported in a study by Ma et al. [50]. This study also found that altered microbiota in first-episode, drug-naive patients correlates with regional grey matter volumes, which suggests a possible link between brain structure and the gut microbiome that may be involved in the pathophysiology of psychosis [50].

**Figure 1:**
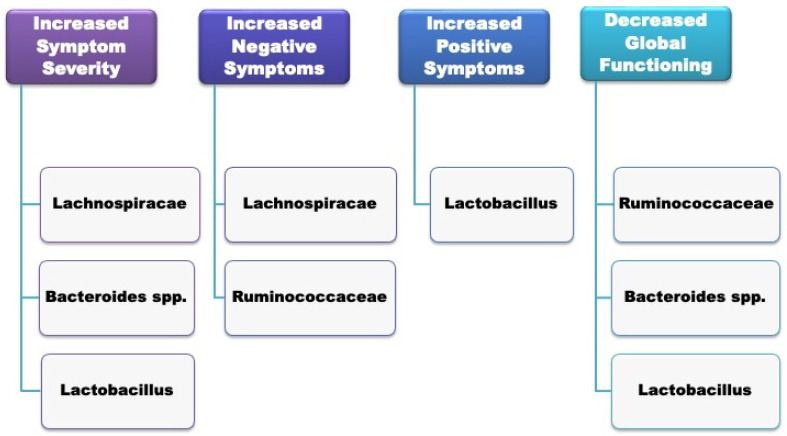
Gut microbiome composition and clinical features of the first episode of psychosis (FEP).

Gut microbial composition has been associated with the severity of psychotic symptoms and a poorer global functioning among patients with first-episode psychosis of any psychiatric cause at the time of hospitalisation [62]. In particular, *Lachnospiraceae*, *Bacteroides* spp. and *Lactobacillus* significantly correlated with increased symptom severity; negative symptoms correlated with *Lachnospiraceae* and *Ruminococcaceae* spps.; increased positive symptoms were associated with *Lactobacillus*; and decreased global functioning correlated with *Ruminococcaceae*, *Bacteroides* and spp. *Lactobacillus*. A summary of the specific microbiome alterations associated with the clinical features of FEP can be seen in Figure 1.

Moreover, the same research brought up important implications regarding the impact of gut microbiome in predicting remission. Treatment response was worse for the patients with the greatest differences in gut microbiota, and remission during a one-year follow-up was substantially less frequent for FEP patients with a higher degree of microbiome abnormalities, compared to patients with a microbiota profile similar to the controls. More specifically, 70% had a remission of symptoms within one year, compared to only 28% with ‘abnormal’ microbiota [62].

Table 2 summarises all the key studies (both experimental and animal) that were included in our review, while also providing additional comments.

## 4. Conclusions and Future Directions

The present review discusses the contributory role of gut microbiome on the brain and behaviours and its effect on the pathophysiology of psychiatric disorders, focusing on schizophrenia and the first episode of psychosis. Despite the inconsistency in microbiome composition changes reported in various studies, the existing evidence strongly suggests significant microbiome alterations in schizophrenic subjects compared to healthy controls and outlines the microbiome’s significant role in the onset, development, pathology, symptom severity, as well as global functioning, disease progression and treatment response in schizophrenia.

The microbiome revolution has opened new frontiers and contributes to a greater understanding of the pathogenesis of neuropsychiatric disorders, promoting precise data-driven diagnostics in psychiatry and novel treatments. It is, however, important to highlight that a significant portion of existing studies are of cross-sectional design, which makes the establishment of causality challenging. In addition, the majority of research on the microbiota–gut–brain axis to date has been conducted on animal models and there are concerns that, for instance, the extrapolation of the findings derived from rodent FMT studies may result in overstating the role of the gut microbiome in human disease [72]. In addition, studies investigating the microbiome as a novel target for mental health disorders are usually underpowered, not addressing microbiome functionality, compositional nature or confounding factors [73], and the meta-analytic findings of the application of several ‘gut biotic’ supplements in psychiatric disorders has not been that impressive to date. Thus, the need for much more human research using data analysis approaches that deal more effectively with these issues [73] is imperative. Further research on the potential role of gut microbiome as a prognostic indicator for high-risk individuals could also be of utmost importance, by identifying susceptible populations who could be provided timely and personalised interventions to prevent further decline.

## Figures and Tables

**Table 1:** Gut microbiome and schizophrenia/first episode of psychosis: changes in microbiome composition and possible pathophysiological mechanisms.

	Microbiome Changes	Pathophysiological Mechanisms
Schizophrenia	No changes in alpha diversity (most studies); Changes in beta diversity (consistent finding); Reduced *Ruminococcus* and *Roseburia*; increased *Veillonella*; Decreased *Proteobacteria*; Increased *Proteobacteria* and *Fusobacteria*; *Firmicutes* decreased; Alterations in *Aerococcaceae*, *Bifidobacteriaceae*, *Brucellaceae*, *Pasteurellaceae* and *Rikenellaceae*; Reduced levels of the phylum *Proteobacteria* and increased *Actinobacteria* and *Bacteroidetes*; Mediating factors: lifestyle changes (e.g., unhealthy diet and smoking), antipsychotic medication and age.	Increased intestinal permeability; Systemic low-level inflammation/immune system involvement/proinflammatory cytokines; Modulation of tryptophan–kynurenine metabolism; Altered amino acid and lipid metabolisms; Disruptions in the glutamate–glutamine–GABA cycle and reduced brain glutamate; Metabolic pathways: vitamin B6, fatty acid, starch and sucrose, cysteine, methionine, linoleic acid metabolism, degradation of xenobiotics; Alterations in brain structure.
First episode of psychosis (FEP)	Relative reduction in alpha diversity; Elevated *Clostridiales*, *Prevotella* and *Lactobacillus ruminis*; Lower numbers of faecal *Bifidobacterium* spp., *Escherichia coli* and *Lactobacillus* spp., and higher numbers of faecal *Clostridium coccoides*; Increased abundance of the harmful phylum *Proteobacteria*; Marked changes in *Christensenellaceae*, *Enterobacteriaceae*, *Pasteurellaceae*, *Turicibacteraceae* at the family level and *Escherichia* at the genus level.	Lower serum tryptophan levels; Higher kynurenic acid (KYNA) levels; Antipsychotic medication induced metabolic changes; Link between gut microbiome and changes in brain structure (grey matter volumes).

**Table 2:** A summary of the key experimental/animal studies included in this review.

First Author/Year	Type of Study	Findings and Comments
Zhu, Guo, 2020 [42]	Animal (mice)	FMT from schizophrenic patients into mice treated with antibiotics resulted in psychomotor hyperactivity and impaired learning and memory. Elevation of the kynurenine–kynurenic acid pathway of tryptophan degradation in both the central and peripheral nervous systems. Elevated basal extracellular dopamine in prefrontal cortex and 5-HT in hippocampus.
Zheng, 2019 [44]	Animal (mice) and Human (schizophrenic vs. controls)	Altered amino acid and lipid metabolisms, along with disruptions in the glutamate–glutamine–GABA cycle and decreased brain glutamate. *Veillonellaceae* OTU191 had a negative correlation with PANNS scores, whereas *Bacteroidaceae* OTU172, *Streptococcaceae* OTU834 and two *Lachnospiraceae* OTUs (477 and 629) had a positive correlation with PANSS scores.
Li, 2021 [49]	Human (schizophrenic vs. controls)	Relative abundance of *Ruminococcus* and *Roseburia* was significantly reduced at the genus level, while the abundance of *Veillonella* was significantly increased in schizophrenic patients compared to the controls.
Nguyen, 2019 [53]	Human (schizophrenic vs. controls)	*Proteobacteria* were relatively lower in schizophrenic subjects compared to the controls. At the genus level, *Anaerococcus* was relatively higher in schizophrenic subjects, whereas *Haemophilus*, *Sutterella* and *Clostridium* were reduced. In schizophrenic patients, the abundance of Ruminococcaceae was associated with reduced severity of negative symptoms. *Bacteroides* was correlated with more severe depressive symptoms.
Shen, 2018 [48]	Human (schizophrenic vs. controls)	Abundance of *Proteobacteria* (at the phylum level) was significantly higher in schizophrenic subjects. At the genus level, the relative abundance of *Succinivibrio*, *Megasphaera*, *Collinsella*, *Clostridium*, *Klebsiella* and *Methanobrevibacter* was significantly increased, while the abundance of *Blautia*, *Coprococcus* and *Roseburia* was reduced. Numerous metabolic pathways were significantly different between the healthy controls and schizophrenic subjects, such as vitamin B6 and fatty acid.
Ma, 2020 [50]	Human (FEP vs. schizophrenic vs. controls)	Both first-episode psychotic (FSCZ) patients, and chronically antipsychotic-treated schizophrenic subjects (TSCZ) had marked changes in gut microbiome composition in certain taxa, including *Christensenellaceae*, *Enterobacteriaceae*, *Pasteurellaceae*, *Turicibacteraceae* at the family level and *Escherichia* at the genus level. Major disturbances in the gut microbiome composition in TSCZ compared to FSCZ patients (eg. *Enterococcaceae* and *Lactobacillaceae*). Certain schizophrenia-related microbiota correlated with the right middle frontal gyrus volume, which was abnormal in schizophrenic subjects.
Pyndt Jørgensen, 2015 [63]	Animal (rats)	Hyperactivity linked to an elevation in *Lachnospiraceae* and *Clostridiaceae*. At the genus level, it was related to increased *Roseburia*, *Clostridium* and *Odoribacter*.
Dunphy-Doherty, 2018 [64]	Animal (rats)	Socially isolated rats had altered microbiota composition with elevated *Actinobacteria* and reduced *Clostridia* class compared to controls. Differences were also observed at the genus level. Positive correlations were seen between microbiota and hippocampal IL-6 and IL-10, conditioned freezing and open-field exploration. Adverse early life stress resulting from continuous social isolation increased ‘anxiety-like’ behaviour and impaired associative learning and memory that went along with alterations to gut microbiota, reduced hippocampal IL-6 and IL-10 and neurogenesis.
He, 2018 [67]	Human (high-risk (HR) subjects vs. ultra-high-risk (UHR) subjects vs. health controls (HC))	Increased *Clostridiales*, *Lactobacillales* and *Bacteroidales* were noted in the faecal samples of UHR subjects compared to the other two groups. Increased production of short-chain fatty acids (SCFAs), as indicated by changes in microbiota composition, which can lead to the activation of microglia and disruption of membrane metabolism.
Yuan, 2018 [68]	Human (FEP vs. healthy controls (HC))	FEP subjects had significantly reduced numbers of faecal *Bifidobacterium* spp., *Escherichia coli* and *Lactobacillus* spp. Significantly higher numbers of faecal *Clostridium coccoides* group in the patient group. After 24-week risperidone treatment, significant increases were noted in body weight, BMI, fasting blood glucose, triglycerides, LDL and a major elevation in the numbers of *fecal Bifidobacterium* spp. and *E. coli*. Additionally, significant decreases in the numbers of faecal *Clostridium coccoides* group and *Lactobacillus* spp.
Zhang, 2018 [69]	Human (schizophrenic (SC) vs. healthy controls (HC))	Increased abundance of harmful bacterial (*Proteobacteria*) and decreased short-chain fatty acid (SCFA)-producing bacteria, such as the *Faecalibacterium* and *Lachnospiraceae* genera in schizophrenic subjects. Relative reduction in alpha diversity and altered composition in the gut mycobiota. Higher levels of *Chaetomium* and a lower level of *Trichoderma* in the schizophrenic group.
Zhu, Ju, 2020 [70]	Human (medication-free psychotic vs. controls) and animals (mice)	Psychotic subjects had a higher alpha diversity and higher beta diversity. Differences in short-chain fatty acid synthesis, tryptophan metabolism and synthesis/degradation of neurotransmitters associated with schizophrenia. FMT of a schizophrenia-enriched bacterium, *Streptococcus vestibularis*, induced deficits in social behaviours and altered neurotransmitter levels in peripheral tissues.
Schwarz, 2018 [62]	Human (FEP vs. healthy controls)	Elevated *Lactobacillus* group bacteria in FEP-subjects that significantly correlated with severity along different symptom domains. FEP subjects with the highest microbiome alterations showed a poorer response after up to 12 months of treatment.

## Data Availability

Not applicable.
